# Editorial: Novel antipsychotics within and beyond clinical trials: The treatment of overlapping psychiatric disorders with D3-D2 partial agonists

**DOI:** 10.3389/fpsyt.2022.1038627

**Published:** 2022-09-28

**Authors:** György Németh, Réka Csehi

**Affiliations:** ^1^Gedeon Richter Plc, Budapest, Hungary; ^2^Institute of Genomic Medicine and Rare Disorders, Semmelweis University, Budapest, Hungary

**Keywords:** overlapping symptoms, psychiatry, transdiagnostic approach, psychopharmacology, dopamine partial agonist, D2-D3 receptors, real-world evidence (RWE), real world data (RWD)

The main taxonomies, the Diagnostic and Statistical Manual of Mental Disorders (currently in 5th ed.) and the International Classification of Diseases (currently in 11th ed.), have an immense impact on how we interpret, appraise and manage psychiatric disorders (1, 2). In these categorical systems, each disease is distinct and appear only once in a clearly defined place (3). However, there seems to be clusters of dimensional symptoms (i.e., differing in severity) that are characteristic of different disorders (3). Therefore, most psychiatric disorders cannot be conceptualized as distinct entities, but rather as complex combinations of dimensional symptoms.

To counter the limitations of the current classification systems, the “transdiagnostic” approach is gaining momentum, capturing the overlap between psychiatric disorders better, therefore enabling novel ways of conceptualizing the underlying theories and mechanisms contributing to mental ill health (4). The goal of the transdiagnostic approach is to allow for the testing, recognition, and utilization of a general theory of psychopathology by aiming to understand the shared, overarching processes that cut across the classification systems. Despite several limitations and unsolved issues, the transdiagnostic research identified groups or patterns of symptoms that cluster together in clinically meaningful ways. This may reflect a common etiological process, course and treatment response, as well as suggests that the co-occurrence of mental disorders might not reflect the co-occurrence of genuinely distinct syndromes—rather, it could be an artifact that emerged from the format of the currently used categorical classification system (3, 5, 6).

Taking on the transdiagnostic approach, the aim of this Research Topic is to examine the role of dopamine partial agonists in the treatment of overlapping psychiatric disorders within and beyond clinical trials. Given the fact that many of the major psychiatric disorders have shared genetic, pathophysiological, as well as environmental elements, there is a high probability that there are common medical solutions too. Based both on the results of several phase II and III clinical trials that have shown efficacy across several neuropsychiatric disorders, as well as the real-world data available investigating the extent to which an intervention influence the clinical picture when provided under usual circumstances of health care practice, there is a high possibility that dopamine partial agonists, such as cariprazine, can be safe and efficacious in a wide variety of symptom clusters.

In this Research Topic, 27 articles were published touching on a broad spectrum of indications, symptoms, and the consequences of dopamine D3 receptor dysfunction at a pathophysiological level.

Despite the benefits of randomized controlled trials, the generation of real-world data, like electronic/medical health records or clinical case reports, is recommended to complement the knowledge gained from clinical trials (7, 8). This way, data can be collected from more heterogenous populations with a wider range of comorbid disorders or adjunctive treatments, therefore increasing the external validity. Firstly, Csehi et al. conducted the first systematic review of cariprazine case reports, showing that cariprazine is effective across many symptoms and many disorders, like schizophrenia, bipolar disorder, adjunctive MDD, borderline personality disorder, obsessive-compulsive disorder or Wernicke-Korsakoff syndrome. Bogren et al. present the case of a patient with long-standing treatment-resistant schizoaffective disorder, where cariprazine in combination with clozapine yielded a near-complete remission of persistent negative and psychosocial issues, therefore improving the patient's quality of life. Vannucchi et al. describe three cases: the first patient had bipolar disorder with cocaine use disorder, the second patient experienced positive and cognitive symptoms, while the third patient suffered from psychosis. In all three cases, cariprazine treatment successfully improved patients' condition. Then, Taube presents a case where the patient with schizophrenia experienced severe side effects of first-generation antipsychotics and was therefore switched to cariprazine. The side-effects subsided and cariprazine provided effective control over the symptoms. The article by Coentre et al. highlights the efficacy and safety of cariprazine in early psychosis. Three cases—including two with comorbid cannabis use—are presented, where patients showed improvements in negative and psychotic symptoms. The case series by Vasiliu describe how cariprazine yielded improvements in negative symptoms and patient functionality in patients with predominant negative symptoms, without causing severe adverse events. Finally, the case report by Cruz et al. shows how cariprazine in combination with quetiapine improved cognitive functioning and negative symptoms, as well as yielded substance abstinence in a patient with comorbid schizophrenia and substance use disorder (SUD). These case reports further support how heterogenic symptoms are within the same diagnosis and show the overlap between symptoms across different diagnoses.

Given the importance of right dosing in achieving effective treatment outcome while minimizing the risk of side-effects, Rancans et al. synthesized data from real-world experience and clinical trials in order to shed light on the appropriate dosing strategies of cariprazine in schizophrenia from treatment initiation through switching strategies to concomitant medications.

Some of the articles cover the topic of schizophrenia with a special focus on negative and cognitive symptoms, as they remain one of the greatest unmet medical needs in its treatment. Mosolov and Yaltonskaya's article offers a comprehensive review of the history and the state-of-the-art of understanding negative symptoms. It strongly raises awareness on the importance of differential diagnosis of primary and secondary negative symptoms, and its methodological and therapeutic implications that involve D2/D3 partial agonism. Demyttenaere et al. conducted a network analysis to better understand the relationship and interactions between different symptoms of a psychiatric disorder by analyzing data from patients with predominant negative symptoms from the cariprazine-risperidone trial (9). According to the findings, depressive and anxious symptoms were the most central symptoms in this patient population, therefore providing important clinical insight. Ivanov et al. present the findings of their observational study, where cariprazine significantly improved predominant negative symptoms in the majority of patients in 4 weeks. In their perspective article, Morozov et al. argue that cariprazine is an adequate pharmacological treatment option for improving social functioning in schizophrenia by reducing negative, cognitive and affective symptoms. Therefore, cariprazine can be viewed as a “socializing drug” that can positively impact on patients' functionality and quality of life. Another aspect for the evaluation of treatment, finding the minimum clinically important difference can be helpful for physicians. Therefore, Czobor et al. aimed at finding this difference at its earliest occurrence in a patient population with predominant negative symptoms, with results suggesting an even lower threshold than previously thought.

In addition, Peris and Szerman review the current advances and future directions in the use of partial agonists in patients with comorbid schizophrenia and SUD, based on the involvement of the dopamine D3 receptors in both disorders.

Bipolar disorder was the focus of three manuscripts. Do et al. provide a comprehensive review of cariprazine in terms of its pharmacological properties, efficacy, and tolerability profile, based on data from clinical trials, including *post-hoc* analyses. The narrative review of Grunze et al. evaluates the potential role of partial agonists in the treatment of addiction in patients with comorbid bipolar disorder given the important role of the dopaminergic system in both disorders: current evidence suggests that partial agonists, especially cariprazine, can indeed improve symptoms associated with substance use and bipolar disorder. Finally, Palacios-Garrán et al. conducted an observational study in which cariprazine in combination with a mood stabilizer (lithium or divalproex) proved to be safe and effective in the treatment of first-episode mania patients, therefore providing the first-ever findings about cariprazine in this patient population.

Further indications have been investigated as well: Mandic-Maravic et al. highlight the unmet need in the treatment of autism spectrum disorder (ASD), with a specific focus on the dopamine theory of the disorder and its potential treatment with dopamine receptor partial agonists. Cariprazine, based on animal model studies and its unique affinity to D3 receptor, may theoretically represent a future opportunity in the treatment of ASD, especially social withdrawal and cognitive symptoms. Molnar et al. summarize the findings from the first-ever study evaluating cariprazine's safety and efficacy on mood and cognitive symptoms in Huntington's disease. They concluded that it is an effective treatment option for this patient population, showing the importance of transdiagnostic approach even in the field of neurology and challenging the terminological discrepancies across the diseases and suggesting common D3 receptor related underlying cross-disease neuropharmacological alterations.

Strengthening this pathophysiological approach, Batinic et al. summarize cariprazine's current therapeutic uses and potential advantages for treating the main symptoms of schizophrenia, bipolar I disorder and MDD and showed that cariprazine may be a drug of choice in patients with predominant negative and cognitive symptoms of schizophrenia, as well as those with metabolic syndrome. Next, in their systematic review and meta-analysis, Dombi et al. provide an updated overview of the evidence behind reduced peripheral levels of BDNF in patients on the schizophrenia-bipolar spectrum as well as evaluate its connection to cognitive symptoms in these disorders.

Although no head-to-head comparisons were conducted for the three partial agonists (cariprazine, aripiprazole and brexpiprazole), using data from controlled studies, meta-analyses and systematic reviews, Mohr et al. evaluated whether the clinical efficacy of these three compounds differs, concluding that these drugs form a heterogenous group, each with its own therapeutic benefit. Milanova et al. present a more detailed and science-based account of the beneficial effect of music therapy on the general wellbeing of patients with schizophrenia by discussing evidence from modern neuroimaging research.

Finally, four articles focus on pharmacology, with an emphasis on the dopamine D3 receptors. In their review, Kiss et al. summarize preclinical and clinical evidence demonstrating that despite many antipsychotics displaying substantial activity for both D2 and D3 receptors *in vitro*, only cariprazine and blonanserin can significantly occupy the D3 receptors *in vivo* and therefore achieve the outcomes associated with D3 activity—although only cariprazine exerts partial agonist effect at these receptors among the two drugs. Hart et al. review the available molecular imaging (PET) studies on the three partial agonists (cariprazine, aripiprazole, brexpiprazole) in order to establish the relationship between plasma concentration of a substance and its binding to the molecular target in the brain. This way, by determining the plasma concentration in individual patients, treatment can be tailored individually. Kehr et al. provide behavioral and *in vivo* neurochemical evidence for the preferential D3 receptor action of cariprazine in rats by comparing the abilities of cariprazine, aripiprazole, and ABT-925 (a selective dopamine D3 antagonist). Moving onto polypharmacy, given its widespread use in clinical practice, the article of Hjorth provides a basis with great visual tools for understanding which antipsychotic combinations are the most optimal based on the drugs' receptor profile.

Overall, the articles of this Research Topic are in line with the view of the transdiagnostic approach whereby there is a substantial overlap between psychiatric illnesses, supporting the notion that these neuropsychiatric disorders should not be conceptualized as separate entities due to the fluidity of diagnostic boundaries. The integration of these data might provide an insight into different indications by showing common underlying neuropharmacological alterations.

The results of these articles are supported by genetic studies that shed light on the polygenic nature of psychiatric illnesses, whereby several common variants with small effects as well as many genetic variants impact on more than one phenotype, implying the existence of the shared genetic etiology of psychiatric disorders (10). In fact, studies have shown that there is a high transcriptome correlation between many disorders, especially schizophrenia, bipolar disorder, ASD and MDD (11).

Dopaminergic dysfunction is well-established in many neurological and psychiatric disorders, including schizophrenia, bipolar disorder, SUD, ASD, addiction and Huntington's disease (12), as demonstrated by the articles of this Research Topic as well. The different dopamine pathways are all involved in neuropsychiatric disorders, and depending on which pathway in what extent is dysregulated, different psychiatric symptoms may arise: hypoactivity in the mesocortical pathway (stemming from the ventral tegmental area, innervating the prefrontal cortex) can mediate negative, cognitive and depressive symptoms; under-activation of dopamine in the mesolimbic pathway (stemming from the ventral tegmental area, innervating the ventral striatum, olfactory tubercule and parts of the limbic system) has been associated with negative symptoms; while overactivation in the associative striatum (nigrostriatal pathway I; originating from the substantia nigra and innervating the associative striatum) has been implied in the development of psychosis (13).

Among the five dopamine receptor types, D2 and D3 receptors play a key role in mediating different psychiatric symptoms. Overactivation of D2 receptors is associated with psychotic and manic symptoms, therefore all antipsychotics target these receptors. On the other hand, D3 receptors have received particular attention due to their anatomical localization: they are prevalently distributed in limbic areas, the hypothalamus, and the ventral tegmental area/substantia nigra and even in prefrontal cortical regions—areas that play a critical role in the regulation of cognition, mood, motivation and negative symptoms (14). Therefore, antipsychotics targeting D3 receptors more potently than the D2 receptors might have potentially favorable effects on these symptoms (15).

Regarding receptor occupancy, for partial agonists to achieve an antipsychotic effect, higher D2 occupancy is required compared to other antipsychotics. In this regard, cariprazine behaves similarly to the other two partial agonists, aripiprazole and brexpiprazole: high D2 receptor occupancy is achieved within a short period of time (16). However, what differentiates cariprazine is that it has the highest affinity to D3 receptors among antipsychotics—even higher than the binding of endogenous dopamine (16). It makes cariprazine the only antipsychotic that is proven to occupy the D3 receptors in the presence of dopamine *in vivo*, exert partial agonist activity here and therefore achieve benefits that might be associated with D3 activity, i.e., improvements in cognitive, affective, and negative symptoms. As these symptoms are characteristic of most mental illnesses and cause the greatest impairment in patient functionality and quality of life, cariprazine is a promising treatment option for a wide variety of neuropsychiatric illnesses, as shown by the articles of this Research Topic as well, offering symptom improvement potentially through restoring altered D3 receptors activity.

## Author contributions

Conceptualization: GN. Both authors participated in the writing, editing, and approved the final version of this manuscript.

## Conflict of interest

Authors GN and RC were employed by Gedeon Richter Plc.

## Publisher's note

All claims expressed in this article are solely those of the authors and do not necessarily represent those of their affiliated organizations, or those of the publisher, the editors and the reviewers. Any product that may be evaluated in this article, or claim that may be made by its manufacturer, is not guaranteed or endorsed by the publisher.
